# Two Diterpenoids and a Cyclopenta[*c*]pyridine Derivative from Roots of *Salvia digitaloids*

**DOI:** 10.3390/ijms150711566

**Published:** 2014-06-27

**Authors:** Shwu-Jen Wu, Chieh-Hung Huang, Yu-Yi Chan, Yu-Ren Liao, Tsong-Long Hwang, Tian-Shung Wu

**Affiliations:** 1 Department of Medical Laboratory Science and Biotechnology, Chung Hwa University of Medical Technology, Tainan 717, Taiwan; 2 Department of Chemistry, National Cheng Kung University, Tainan 701, Taiwan; E-Mails: hjh2000.tw@gmail.com (C.-H.H.); truthloveroy@yahoo.com.tw (Y.-R.L.); 3 Department of Biotechnology, Southern Taiwan University of Science and Technology, Tainan 710, Taiwan; E-Mail: yuyichan@mail.stust.edu.tw; 4 Graduate Institute of Natural Products, College of Medicine, Chang Gung University, Taoyuan 333, Taiwan; E-Mail: htl@mail.cgu.edu.tw

**Keywords:** *Salvia digitaloids*, *Labiatae*, diterpenoid, cyclopenta[*c*]pyridine

## Abstract

Two new glucosides, salviadigitoside A (**1**) and salviatalin A-19-*O*-β-glucoside (**2**), belonging to the salviatalin type diterpenoids, and a new cyclopenta[*c*]pyridine, salviadiginine A (**3**), were isolated from the roots of *Salvia digitaloids*. Structures of these compounds were determined on the basis of spectroscopic analysis. In addition, compounds **1**–**3** were evaluated for their anti-inflammatory activity, but the results showed a weak anti-inflammatory activity.

## 1. Introduction

*Salvia*, the largest genus of the family Labiatae [1], is economically important because it has exhibited various biological and pharmacological activities including antitumor [2], antiallergic [3], antioxidant [4], antimicrobial [5], and antiplatelet aggregation activities [6]. The diterpenoid constituents of this genus are of great interest due to their diverse structures and significant biological activities [7,8,9]. We previously reported a new antitumor agent, neo-tanshinlactone, which was isolated from *Salvia miltiorrhiza* [9], and several notable new abietane diterpene alkaloids from *Salvia yunnanensis* [10].

**Figure 1:**
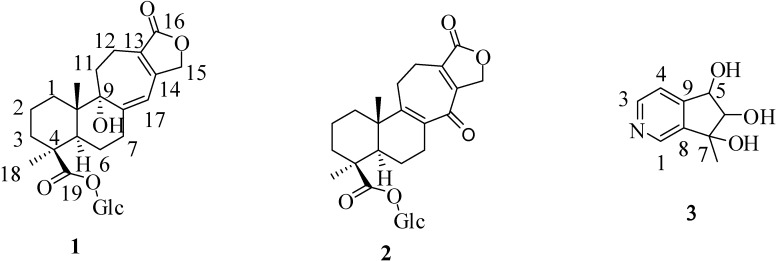
Structure of three new compounds **1**–**3**.

*Salvia digitaloides* is a herbaceous perennial shrub native to the Chinese provinces of Guizhou, Sichuan, and Yunnan, which has been used in traditional Yunnan medicine. The local Tibetans soak the roots of this plant in alcohol to manufacture a special traditional health drink, to make them physically strong. As far as we know, fifteen diterpenoids have been reported from the roots of this plant [11,12]. In our previous study, two diterpenes with novel skeletons were isolated from the chloroform fraction of the roots of *S. digitaloides*, and salviatalin A showed a potent inhibitory effect on superoxide anion production in *N*-formyl-l-methionyl-l-leucyl-l-phenylalanine/cytochalasin B (FMLP/CB)-activated human neutrophils, as well as other anti-inflammatory effects [11]. In order to explore the anti-inflammatory effects and the diterpenoids constituents of the roots of *S. digitaloides* with novel structures, we continued to study the constituents of the *n*-butanol fraction of this plant. In this paper, we report the isolation and structural determination of two new diterpenoid glucosides salviadigitoside A (**1**) and salviatalinA-19-*O*-β-glucoside (**2**) together with a new cyclopenta[*c*]pyridine, salviadiginine A (**3**) (Figure 1) from the roots of *S. digitaloids*. In addition, the inhibitory activites on superoxide generation and elastase release by neutrophils of compounds **1**–**3** were also examined.

## 2. Results and Discussion

### 2.1. Purification and Characterization

The methanol-soluble extract of the dried roots of *S. digitaloides* was suspended in water and then extracted with chloroform and *n*-BuOH successively. The *n*-BuOH fraction was subjected to purification by a combination of conventional chromatographic techniques and resulted in the isolation of three new compounds (**1**–**3**).

### 2.2. Structural Elucidation of Compounds **1**–**3**

**Figure 2:**
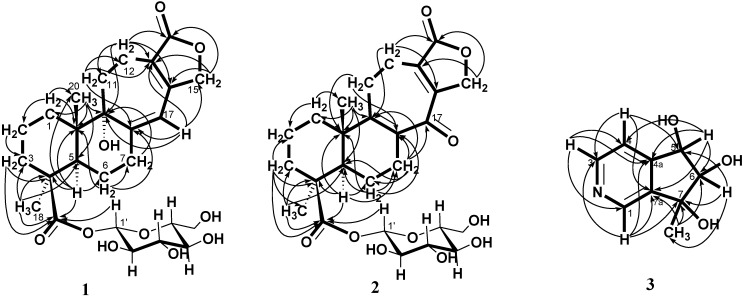
HMBC correlations of **1**−**3**.

Salviadigitoside A (**1**) was obtained as a colorless syrup and had a postitive rotation (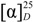 + 56.3, *c* 0.19, MeOH). The high resolution electrospray ionization mass spectroscopy (HRESI-MS) of **1** showed an ion peak at *m*/*z* 531.2209, which was consistent with the molecular formula of C_26_H_36_O_10_Na. This formula implied nine degrees of unsaturation. The UV spectrum of **1** showed absorption maxima at 220 and 283 nm, indicating the presence of α,β,γ,δ-unsaturated ketone moiety in the molecule. The IR spectrum showed strong absorption peaks for OH (3387 cm^−1^), carbonyl group (1728 cm^−1^) and furan ring (763 cm^−1^). The proton signals for an anomeric proton at δ 5.46 (d, *J* = 8.0 Hz), oxygenated methylene at δ 3.82 (1H, dd, *J* = 11.6, 1.4 Hz) and 3.69 (1H, dd, *J* = 11.6, 4.0 Hz), and oxygenated methines at δ 3.45–3.30 (4H, m) suggested the presence of one sugar moiety. In the ^13^C NMR spectrum (Table 1) of **1**, the carbon resonances at δ 95.5, 74.1, 78.5, 71.1, 78.8, 62.4 further confirmed that **1** was substituted with one glucose [13]. Furthermore, the ^13^C NMR spectrum showed 20 remaining carbon signals, including a vinyl carbon (δ 117.0), three quaternary olefinic (δ 160.4, 157.4 and 129.9), two carbonyl (δ 177.8 and 176.7), two quaternary (δ 46.2 and 45.8), one oxygenated quaternary (δ 81.4), one methine (δ 49.0), eight methylene (δ 72.4, 38.7, 38.1, 36.3, 33.9, 26.5, 20.9, and 19.4), and two methyl (δ 29.6 and 16.8) carbons. These results were confirmed by the heteronuclear singular quantum correlation (HSQC) spectrum. The ^1^H NMR spectrum of **1** (Table 1) showed signals for two methyls (δ 1.32 and 0.79) and an oxygen-bearing methylene (H-15, δ 4.77), which are the typical lactone protons. The correlation spectroscopy (COSY) spectrum showed the following proton–proton cross-peaks: H-11 (δ 1.85 and 2.15) to H-12 (δ 2.30 and 2.46), H-6 (δ 2.04) to H-7 (δ 2.30 and 2.91)/H-5, (δ 2.25), and H-2 (δ 1.55, 1.96) to H-1 (δ 1.53, 1.79)/H-3 (δ 1.14, 2.19). The seven-membered C-ring was established by the correlations of H-11 (δ 1.85) with C-8 (δ 160.4), C-12 (δ 19.4) and C-13 (δ 129.9), of H-12 (δ 2.30, 2.46) with C-9 (δ 81.4), C-11 (δ 36.3), C-13 (δ 129.9) and C-14 (δ 157.4), and of H-17 (δ 6.05) with C-8 (δ 160.4), C-9 (δ 81.4) and C-13 (δ 129.9) in the heteronuclear multiple bond correlation (HMBC) spectrum (Table 1, Figure 1). The HMBC spectrum of **1** also showed the conjugated cross-peaks of H-15 (δ 4.77) to C-13 (δ 129.9)/C-14 (δ 157.4)/C-16 (δ 176.7), H-11 (δ 1.85) to C-8 (δ 160.4)/C-13 (δ 129.9) and H-17 (δ 6.05) to C-8 (δ 160.4)/C-9 (δ 81.4)/C-13 (δ 129.9)/C-15 (δ 72.4), indicating that **1** was a γ-lactone ring α,β-fused to a novel 6/6/7 tricyclic-skeleton diterpene containing two double bond conjugated with the carbonyl group. The ^3^*J* HMBC correlations of the methyl protons (H-18) at δ 1.32 with C-3 (δ 38.7), C-4 (δ 45.8), C-5 (δ 49.0), and C-19 (δ 177.8) indicated that the quaternary C-4 was substituted with both methyl and carboxylic acid groups. Moreover, the correlation of the anomeric proton at δ 5.46 with C-19 (δ 177.8) in HMBC spectrum suggested that the glucose was connected to the C-19 carboxylic acid group. In addition, the ^3^*J* HMBC correlations of the oxygenated quaternary carbon (C-9) at δ 81.4 with H-7 (δ 2.30), H-12 (δ 2.30), H-17 (δ 6.05), and H-20 (δ 0.79) showed that the C-9 was substituted with a hydroxyl group. The stereochemistry was confirmed by a nuclear overhauser enhancement spectroscopy (NOESY) experiment, which showed correlations of H-5/Me-18, H-5/H-6α, Me-20/H-2β, Me-20/H-6β, and Me-20/H-11β (Figure 2). Thus, the methyl substituents at C-4 and C-10 have the α- and β-orientation, respectively. Based on the above-mentioned observations, the structure of salviadigitoside A was assigned as **1**.

**Table 1:** ^1^H and ^13^C NMR spectral data and heteronuclear multiple bond correlation (HMBC) correlations for compounds 1 and 2 in CD3OD.

Compound	1				2			
	δ_H_ (*J* in Hz)	δ_C_		HMBC	δ_H_ (*J* in Hz)		δ_C_	HMBC
**1**	β (eq): 1.53 m;	33.9		C-2, C-10,	α (ax): 1.26 td (13.9, 3.8)		37.1	C-2, C-20
	α (ax): 1.79 td (12.8, 3.2)			C-20	β (eq): 2.11 m			
**2**	β (ax): 1.55 m;	20.9		C-3	α (eq): 1.61 dq' (13.9, 3.8)		20.5	
	α (eq): 1.96 dq' (13.6, 3.2)				β (ax): 2.05 m			
**3**	α (ax): 1.14 td (13.6, 3.2);	38.7		C-4, C-19	α (ax): 1.12 td (13.9, 3.8)		38.3	C-2, C-4,
	β (eq): 2.19 m				β (eq): 2.27 dt (13.9, 3.8)			C-18, C-19
**4**		45.8					45.3	
**5**	α (ax): 2.25 d (9.6)	49.0		C-4, C-6,	α (ax): 1.44 d (13.2)		54.4	C-4, C-6, C-7,
				C-10, C-20				C-10, C-19
**6**	2.04 m (2H)	26.5		C-7, C-8, C-10	β (ax): 1.83 qd (13.2, 6.0)		21.1	C-5, C-7,
					α (eq): 2.17 dd (13.2, 6.0)			C-8, C-10
**7**	β (eq): 2.30 m;	38.1		C-9	α (ax): 2.02 td (13.2, 6.0)		29.0	C-5, C-6, C-8,
	α (ax): 2.91 m				β (eq): 2.69 dd (13.2, 6.0)			C-9, C-17
**8**		160.4					137.3	
**9**		81.4					159.3	
**10**		46.2					43.1	
**11**	β (ax): 1.85 t (13.6);	36.3		C-8, C-12,	2.59 m (2H)		27.4	C-8, C-10,
	α (eq): 2.15 m			C-13				C-13
**12**	α (ax): 2.30 m;	19.4		C-9, C-11, C-13, C-14, C-16	α (ax): 2.40 m		24.6	C-9, C-13,
	β (eq): 2.46 dd (16.2, 6.6)				β (eq): 2.59 m			C-16
**13**		129.9					139.9	
**14**		157.4					154.1	
**15**	4.77 m (2H)	72.4		C-13, C-14,	4.84 dd (17.4, 2.4)		71.4	C-14, C-16
				C-16	4.98 dt (17.4, 2.4)			
**16**		176.7					176.0	
**17**	6.05 s	117.0		C-8, C-9,			192.1	
				C-13, C-15				
**18**	α (eq): 1.32 s	29.6		C-3, C-4,	α (eq): 1.31 s		28.7	C-3, C-4,
				C-5, C-19				C-5, C-19
**19**		177.8					177.6	
**20**	β (ax): 0.79 s	16.8		C-1, C-5,	β (ax): 1.04 s		17.8	C-1, C-5,
				C-9, C-10				C-9, C-10
**1'**	5.46 d (8.0)		95.5	C-19		5.48 d (7.9)	95.6	C-19
**2'**	3.30–3.45 m		74.1			3.32–3.42 m	74.2	
**3'**	3.30–3.45 m		78.5 *			3.32–3.42 m	78.4	
**4'**	3.30–3.45 m		71.1			3.32–3.42 m	71.1	
**5'**	3.30–3.45 m		78.8 *			3.32–3.42 m	78.7	
**6'**	3.69 dd (11.6, 4.0)		62.4			3.68 dd (11.8, 4.0)	62.4	
	3.82 dd (11.6, 1.4)					3.80 d (11.8)		

* Assignments may be interchangeable; “m” means overlapping or irresolvable peak.

**Figure 3:**
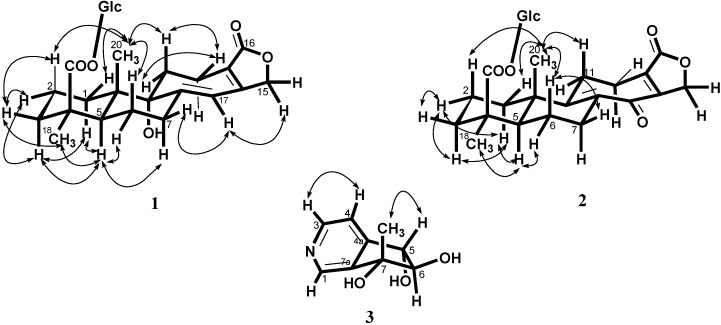
Nuclear overhauser enhancement spectroscopy (NOESY) correlations of **1**–**3**.

SalviatalinA-19-*O*-β-glucoside (**2**) was also obtained as colorless needles with positive rotation (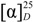 + 107.8, *c* 0.47, MeOH). The HRESI-MS of **2** showed a ion peak at *m*/*z* 529.2053, consistent with the molecular formula of C_26_H_34_O_10_Na, implied ten degrees of unsaturation. The UV spectrum showed an absorption maximum at 251 and 313 nm, and the IR spectrum showed strong absorptions at 3383 and 1751 cm^−1^ for hydroxyl and carbonyl group in the molecule, respectively. The structure of **2** was similar to that of **1**, according to the ^1^H NMR and ^13^C NMR spectral data (Table 1). The differences observed in the ^1^H NMR and ^13^C NMR spectra of **2** compared with those of **1** were occurrence of signals for C-8 and C-9 at δ 137.3 and 159.3 instead of δ 160.4 and 81.4, respectively. In addition, the H-17 signal in **2** disappeared and the carbonyl signal at δ 117.0 in **1** shifted to δ 192.1 in **2**, suggesting that the seven-membered C-ring of **2** was formed the α,β-unsaturated ketone group. Therefore, the structure of salviatalinA-19-*O*-β-glucoside was assigned as **2**, which was supported by COSY, HSQC, HMBC (Table 1, Figure 1), and NOESY (Figure 3) experiments.

**Table 2:** ^1^H and ^13^C NMR spectral data and HMBC correlations for compound **3** in CD_3_OD.

Compound	3		
	δ_H_ (*J* in Hz)	δ_C_	HMBC
**1**	8.53 s	145.4	C-3, C-4a, C-7, C-7a
**3**	8.48 d (5.1)	149.4	C-1, C-4, C-4a
**4**	7.43 d (5.1)	120.4	C-3, C-5, C-7a
**4a**		151.1	
**5**	4.68 d (7.7)	76.8	C-4, C-4a, C-6, C-7a
**6**	4.04 d (7.7)	90.3	C-5, C-7, 7-CH_3_
**7**		78.2	
**7-CH_3_**	1.36 s	23.1	C-6, C-7, C-7a
**7a**		143.2	

Salviadiginine A (**3**) was obtained as colorless needles, 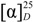 + 15.5 (*c* 0.09, MeOH). The molecular formula, C_9_H_11_NO_3_, was established by HRESI-MS (182.0816 [M + H]^+^, calcd. for 182.0817), implying five degrees of unsaturation. The IR spectrum displayed absorption characteristic of hydroxyl and aromatic groups (3321 and 1608 cm^−1^), and the UV spectrum showed absorbance at λ_max_ 259 nm. The presence of α,β,γ-disubstituted pyridine was revealed by ^1^H NMR signals at δ 7.43 (d, *J* = 5.1 Hz, H-4), 8.48 (d, *J* = 5.1 Hz, H-3) and 8.53 (s, H-1), together with ^13^C NMR signals at δ 120.4 (d), 143.2 (s), 145.4 (d), 149.4 (d), and 151.1 (s). The signals at δ 76.8 (d) and 90.3 (d) in the ^13^C NMR spectrum, as well as a resonance at δ 4.04 (1H, d, *J* = 7.7 Hz) and 4.68 (1H, d, *J* = 7.7 Hz) in the ^1^H NMR spectrum, provided evidence for the presence of the fragment –CH(OH)–CH(OH)– in **3**. In the HMBC experiment (Table 2, Figure 1) of **3**, correlations of H-5 (δ 4.68)/C-4 (δ 120.4), C-4a (δ 151.1), C-6 (δ 90.3), and C-7a (δ 143.2), and of the methyl group (δ 1.36)/C-6 (δ 90.3), C-7 (δ 78.2), and C-7a (δ 143.2) suggested that the planar structure of salviadiginine A was established as **3**. The relative stereochemistry of three hydroxyls located toward pseudo equatorial orientation was due to the presence of nuclear overhauser effect (NOE) correlation between H-5 and 7-CH_3_ and the absence of NOE correlation between H-5/7-CH_3_ and H-6 in the NOESY spectrum (Figure 3). The absolute configuration of the 5,6,7-triol moiety was determined by application of the circular dichroic (CD) exciton chirality method [14]. The negative Cotton effect at 225 nm (Δε −1.19), the positive Cotton effect at 268 nm (Δε +2.19) and the negative Cotton effect at 291 nm (Δε −0.12) were correlated to the 5*S*, 6*R*, 7*R* configuration. Thus, compound **3** was determined to be (5*S*,6*R*,7*R*)-6,7-dihydro-7-methyl-5*H*-cyclopenta[*c*]pyridine-5α,6α,7α-triol.

### 2.3. Anti-Inflammatory Activity

Neutrophils are the most abundant white blood cells in human-bodies. They play an important role in the immune defense against various diseases, and they also participate in the development of the inflammatory reaction. As a response to different stimuli, activated neutrophils secrete a series of cytotoxins, such as the superoxide anion radical (O^2−^), a precursor to other reactive oxygen species (ROS), bioactive lipids, granule proteases, and neutrophil elastase, a major contributor to destruction of tissue in chronic inflammatory disease. Suppression of the extensive or inappropriate activation of neutrophils by drugs has been suggested as a way to improve inflammatory diseases [15,16,17,18]. In the anti-inflammatory assay (Table 3), neutrophils were activated by *N*-formyl-l-methionyl-l-leucyl-l-phenylalanine/cytochalasin B (FMLP/CB) to produce superoxide anion and elastase, which are both mediators of neutrophilic inflammation. In this study, the percentage of inhibition (Inh %) for compounds **1**–**3** at 10 μM concentration against FMLP/CB-induced superoxide anion generation by human neutrophils were 11.14 ± 3.27, 9.35 ± 4.85 and 7.88 ± 5.53, respectively. In addition, the percentage of inhibition (Inh %) for compounds **1**–**3** at 10 μM concentration in the FMLP/CB-induced elastase release assay were 11.83 ± 6.06, 11.12 ± 2.76 and 2.83 ± 4.91, respectively.

**Table 3:** Effects of pure compounds on superoxide anion generation and elastase release in FMLP/CB-induced human neutrophils.

Compound	Superoxide Anion	Elastase Release
	Inh %	Inh %
**1**	11.14 ± 3.27 *	11.83 ± 6.06
**2**	9.35 ± 4.85	11.12 ± 2.76 *
**3**	7.88 ± 5.53	2.83 ± 4.19
LY294002 ^a^	1.0± 0.3	1.5± 0.4

Percentage of inhibition (Inh %) at 10 mM concentration. Results are presented as mean ± SEM (*n* = 3); * *p* < 0.05 compared with the control value (DMSO); ^a^, this phosphatidylinositol-3-kinase inhibitor was used as a positive control for inhibition of superoxide anion generation and elastase release (*n* = 3).

## 3. Experimental Section

### 3.1. General

All the chemicals were purchased from Merck KGaA (Darmstadt, Germany) unless specifically indicated. Melting points of purified compounds were determined by a Yanagimoto MP-S3 melting point measuring apparatus without correction. UV spectra were obtained on a Hitachi UV-3210 spectrophotometer (Hitachi, Tokyo, Japan). IR spectra were recorded on a Shimadzu FTIR spectrometer Prestige-21 (Shimadzu, Tokyo, Japan). Optical rotations were measured using a Jasco DIP-370 Polarimeter. Electrospray ionization (ESI) and HRESI mass spectra were recorded on a Bruker APEX II mass spectrometer (Bruker, Rheinstetten, Germany). The NMR spectra, including ^1^H NMR, ^13^C NMR, COSY, NOESY, HMBC, and HSQC experiments, were recorded on Bruker Avance 400 and AV-500 NMR spectrometers (Bruker, Rheinstetten, Germany) with TMS as the internal reference, and chemical shifts are expressed in δ (ppm). Silica gel (Merck, Germany, 70–230, 230–400 mesh) was used for column chromatography and thin layer chromatography (TLC) was conducted on pre-coated Kiesel gel 60 F_254_ plates (Merck), and the spots were visualized by UV.

### 3.2. Plant Materials

The roots of *S. digitaloides* were collected from in Li Jiang, Yunnan Province, People’s Republic of China, in October 2004 by S. Zhang, Institute of Materia Medica, Chinese Academy of Medicinal Sciences, Beijing, China, and identified by Kuoh, C.S. Department of Life Sciences, National Cheng Kung University, Tainan, Taiwan. Permission was obtained to export the plant material from the China into Taiwan. A voucher specimen (TSWu-20041015) was deposited at the Herbarium of National Cheng Kung University.

### 3.3. Extraction and Isolation

The dried roots of *S. digitaloides* (3.0 kg) were pulverized into powder and extracted six times with methanol under reflux. The methanol soluble extract was concentrated under reduced pressure to give dark brown syrup (220 g). The methanol-soluble extract was suspended in water and then extracted with chloroform and *n*-BuOH successively to afford the chloroform layer (70 g), *n*-BuOH fraction (68 g). The *n*-BuOH fraction was chromatographed over reversed-phase Diaion HP-20 gel using water and methanol gradients (water:methanol = 1:0, 9:1, 5:1, 3:1, 2:1, 1:1, 1:3, 1:9, 0:1) and afforded 8 fractions according to the TLC monitoring. Fraction (Fr.) 3 was chromatographed on silica gel, eluted with a mixture of chloroform and methanol (3:1) to give five subfractions (Fr. 3.1–3.5). Fr. 3.3 was subjected to silica gel column chromatography (CC) eluted by solvent mixture of chloroform and methanol (5:1) to yield salviatalin A-19-*O*-β-glucoside (**2**) (4.7 mg). Fraction 5 was purified by silica gel CC with a mixing eluent of chloroform and methanol (5:1) to afford six subfractions (Fr. 5.1–5.6). Fr. 5.2 was further purified by silica gel CC and successive preparative thin layer chromatography (pTLC) eluted by chloroform and methanol (5:1) to produce salviadigitoside A (**1**) (2.1 mg). Fraction 6 was subjected to silica gel CC eluted by solvent mixture of chloroform and methanol (5:1) and all the collections were combined to seven subfractions (Fr. 6.1–6.7) according to the TLC monitoring. Fr. 6.5 was subjected to silica gel CC eluted by solvent mixture of chloroform and methanol (5:1) and the successive pTLC purification to yield salviadiginine A (**3**) (5.1 mg).

#### 3.1.1. Salviadigitoside A (**1**)

Colorless syrup; 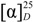 + 56.3 (*c* 0.19, MeOH); UV (MeOH), λ_max_ (log ε) 220 (3.60), 283 (3.90) nm; IR (KBr) ν_max_ cm^−1^: 3387, 1728, 763 cm^−1^; ^1^H and ^13^C NMR see Table 1; electrospray ionization mass spectroscopy (ESI-MS) *m*/*z* (rel. int.): 531 [M + Na]^+^; HRESI-MS *m*/*z*: 531.2209 [M + Na]^+^ (calcd. for C_26_H_36_O_10_Na, 531.2206).

#### 3.1.2. SalviatalinA-19-*O*-β-glucoside (**2**)

Colorless needles (from MeOH); mp, 172–175 °C; 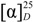 + 107.8 (*c* 0.47, MeOH ); UV (MeOH), λ_max_ (log ε) 251 (3.92), 313 (3.51) nm; IR (KBr) ν_max_ cm^−1^: 3383, 1751, 759 cm^−1^ ; ^1^H and ^13^C NMR see Table 1; ESI-MS *m*/*z* (rel. int.): 529 [M + Na]^+^; HRESI-MS *m*/*z*: 529.2053 [M + Na]^+^ (calcd. for C_26_H_34_O_10_Na, 529.2050).

#### 3.1.3. Salviadiginine A (**3**)

Colorless needles (from MeOH); mp, 194–196 °C; 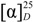 + 15.5 (*c* 0.09, MeOH); UV (MeOH) λ_max_ (log ε) nm: 259 (3.46), 265 (3.38) nm; IR (KBr) ν_max_ cm^−1^: 3321 cm^−1^; ^1^H and ^13^C NMR see Table 2; ESI-MS *m*/*z* (rel. int.): 182 [M + H]^+^; HRESI-MS *m*/*z*: 182.0816 [M + H]^+^ (calcd. for C_9_H_12_NO_3_, 182.0817); CD (MeOH, 2.76 × 10^−4^ M) (Δε) 291 (−0.12), 268 (2.19), 225 (−1.19) nm.

### 3.2. Biological Assay

#### 3.2.1. Preparation of Human Neutrophils

Whole blood was obtained from healthy donors (20–30 years old) by venipuncture. Human neutrophils were isolated by means of dextran sedimentation, Ficoll-Paque centrifugation, and hypotonic lysis of erythrocytes [19]. The purified neutrophils were resuspended in a Ca^2+^-free Hank’s balanced salt solution buffer at pH 7.4 and kept at 4 °C before use.

#### 3.2.2. Measurement of Superoxide Anion Generation

The superoxide anion generation assay was carried out according to an established protocol [20], which monitors the reduction of ferricytochrome c inhibited by superoxide dismutase. In brief, the neutrophils were equilibrated with 0.5 mg/mL ferricytochrome c and 1 mM Ca^2+^ at 37 °C for 2 min and then incubated with each test compound for 5 min. Cells were incubated with cytochalasin B (CB, 1 μg/mL) for 3 min, before activation by formyl-l-methionyl-l-leucyl-l-phenylalanine (FMLP, 100 nM) for 10 min. FMLP/CB was used as a stimulant to activate neutrophils to produce superoxide anion. The changes in absorbance with reduction of ferricytochrome c at 550 nm were continuously monitored in a double-beam, six-cell positioned spectrophotometer (Hitachi U-3010, Tokyo, Japan) with constant stirring. Calculations were based on differences in the reactions with and without superoxide dismutase (100 U/mL) divided by the extinction coefficient for the reduction of ferricytochrome c (ε = 21.1/mM/10 mm). LY294002 was used as a positive control.

#### 3.2.3. Elastase Release Assays.

Elastase release was measured by degranulation of azurophilic granules in human neutrophils as described previously [21]. Neutrophils were equilibrated in an elastase substrate, MeO-Suc-Ala-Ala-Pro-Val-*p*-nitroanilide (100 μM), at 37 °C for 2 min and then incubated with test compounds for 5 min. Cells were activated by 100 nM FMLP and 0.5 μg/mL CB, and the changes in absorbance at 405 nm were monitored continuously to assay elastase release. The results were expressed as the percentage of elastase release in the FMLP/CB-activated, drug-free control system. LY294002 was used as a positive control.

## 4. Conclusions

**Scheme 1:**
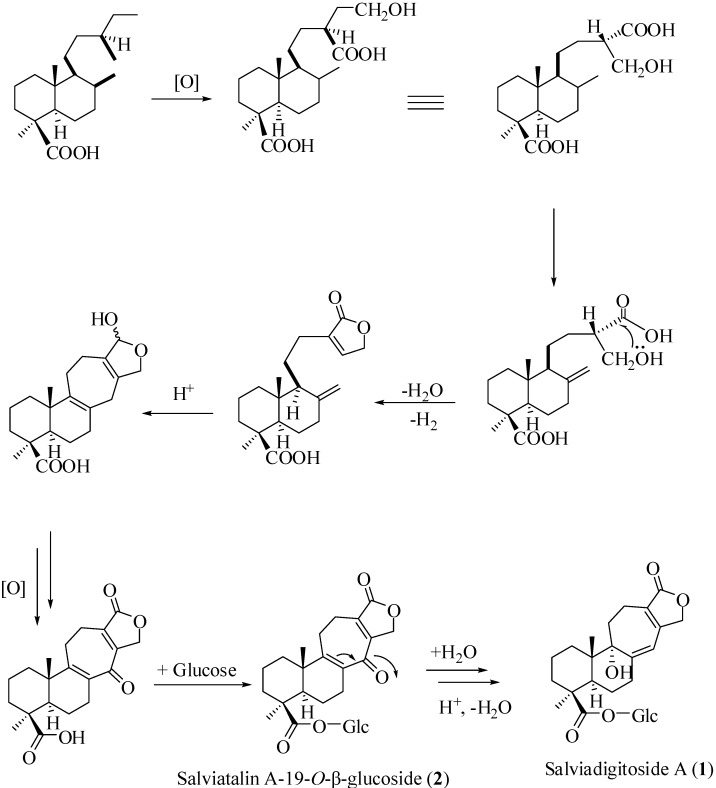
Plausible biosynthetic pathway of compounds **1** and **2**.

In summary, three new compounds were characterized from the rhizomes the *S. digitaloides*. These compounds could be classified into two basic skeletons. Salviadigitoside A (**1**) and salviatalinA-19-*O*-β-glucoside (**2**) were belonging to the salviatalin type diterpenoids of natural origin. The biosynthetic pathway to both compounds **1**–**2** is illustrated in Scheme 1. Salviadiginine A (**3**) was identified to possess a cyclopenta[*c*]pyridine-type skeleton. Compounds **1**–**3** were examined for their inhibitory effects of superoxide anion generation and elastase release by human neutrophils in response to *N*-formyl-l-methionyl-l-leucyl-l-phenylalanine/cytochalasin B (FMLP/CB). The results revealed these compounds have only weak anti-inflammatory activity.
